# Personal

**Published:** 1902-06

**Authors:** 


### PERSONAL.

Dr. Edward J. Bodenbender, of 660 Walden Avenue, has been appointed East Side Homeopathic District Physician, to succeed the late Dr. E. A. Fisher. Dr. Bodenbender was appointed from the civil service list, on which he had a marking of 100 per cent.

Dr. H. R. Gaylord, of Buffalo, pathologist at the state laboratory, will sail for Europe about July 1, to be absent a year or more pursuing investigations in the surgical clinics of Germany. He will be accompanied by Mrs. Gaylord.

Dr. L. J. McAdam, of Buffalo, announces that hereafter he will be professionally associated with Dr. C. M. Daniels, 315 Jersey Street, corner of Plymouth Avenue. Hours: 9 to 11 a.m.; 2 to 4 p.m. Telephone, Tupper 336.

Dr. Charles F. Howard, of Buffalo, has been elected president of the board of managers of the New York State Reformatory located at Elmira.

Dr. Max C. Breuer, of Buffalo, sails for Europe late in June to spend the remainder of the summer in Germany.

Dr. James Stoddart, of Buffalo, has removed from 90 York Street to 770 Elmwood Avenue.

Dr. Myrtle A. Hoag, of Buffalo, has removed her offices from 397 to 426 Dearborn Street.

